# The use of a clinical decision support tool to assess the risk of QT drug–drug interactions in community pharmacies

**DOI:** 10.1177/2042098621996098

**Published:** 2021-02-24

**Authors:** Florine A. Berger, Heleen van der Sijs, Teun van Gelder, Patricia M. L. A. van den Bemt

**Affiliations:** Department of Hospital Pharmacy, Erasmus University Medical Centre, Department of Hospital Pharmacy, Doctor Molewaterplein 40, 3015 GD Rotterdam, The Netherlands; Department of Hospital Pharmacy, Erasmus University Medical Centre, Rotterdam, The Netherlands; Department of Hospital Pharmacy, Erasmus University Medical Centre, Rotterdam, The Netherlands; Department of Clinical Pharmacy and Toxicology, Leiden University Medical Centre, Leiden, The Netherlands; Department of Hospital Pharmacy, Erasmus University Medical Centre, Rotterdam, The Netherlands; Department of Clinical Pharmacy and Pharmacology, University Medical Centre Groningen, Groningen, The Netherlands

**Keywords:** clinical decision support systems, community pharmacies, drug–drug interactions, intervention, primary care

## Abstract

**Introduction:**

The handling of drug–drug interactions regarding QTc-prolongation (QT-DDIs) is not well defined. A clinical decision support (CDS) tool will support risk management of QT-DDIs. Therefore, we studied the effect of a CDS tool on the proportion of QT-DDIs for which an intervention was considered by pharmacists.

**Methods:**

An intervention study was performed using a pre- and post-design in 20 community pharmacies in The Netherlands. All QT-DDIs that occurred during a before- and after-period of three months were included. The impact of the use of a CDS tool to support the handling of QT-DDIs was studied. For each QT-DDI, handling of the QT-DDI and patient characteristics were extracted from the pharmacy information system. Primary outcome was the proportion of QT-DDIs with an intervention. Secondary outcomes were the type of interventions and the time associated with handling QT-DDIs. Logistic regression analysis was used to analyse the primary outcome.

**Results:**

Two hundred and forty-four QT-DDIs pre-CDS tool and 157 QT-DDIs post-CDS tool were included. Pharmacists intervened in 43.0% and 35.7% of the QT-DDIs pre- and post-CDS tool respectively (odds ratio 0.74; 95% confidence interval 0.49–1.11). Substitution of interacting agents was the most frequent intervention. Pharmacists spent 20.8 ± 3.5 min (mean ± SD) on handling QT-DDIs pre-CDS tool, which was reduced to 14.9 ± 2.4 min (mean ± SD) post-CDS tool. Of these, 4.5 ± 0.7 min (mean ± SD) were spent on the CDS tool.

**Conclusion:**

The CDS tool might be a first step to developing a tool to manage QT-DDIs *via* a structured approach. Improvement of the tool is needed in order to increase its diagnostic value and reduce redundant QT-DDI alerts.

**Plain Language Summary:**

**The use of a tool to support the handling of QTc-prolonging drug interactions in community pharmacies**

**Introduction:** Several drugs have the ability to cause heart rhythm disturbances as a rare side effect. This rhythm disturbance is called QTc-interval prolongation. It may result in cardiac arrest. For health care professionals, such as physicians and pharmacists, it is difficult to decide whether or not it is safe to proceed treating a patient with combinations of two or more of these QT-prolonging drugs. Recently, a tool was developed that supports the risk management of these QT drug–drug interactions (QT-DDIs).

**Methods:** In this study, we studied the effect of this tool on the proportion of QT-DDIs for which an intervention was considered by pharmacists. An intervention study was performed using a pre- and post-design in 20 community pharmacies in The Netherlands. All QT-DDIs that occurred during a before- and after-period of 3 months were included.

**Results:** Two hundred and forty-four QT-DDIs pre-implementation of the tool and 157 QT-DDIs post-implementation of the tool were included. Pharmacists intervened in 43.0% of the QT-DDIs before the tool was implemented and in 35.7% after implementation of the tool. Substitution of one of the interacting agents was the most frequent intervention. Pharmacists spent less time on handling QT-DDIs when the tool was used.

**Conclusion:** The clinical decision support tool might be a first step to developing a tool to manage QT-DDIs *via* a structured approach.

## Introduction

Drug–drug interactions (DDIs) regarding QTc-prolongation are common in daily practice due to the high number of drugs known for prolonging the QTc-interval. Currently, over 50 drugs are associated with causing Torsade de Points (TdP) by prolonging the QTc-interval, according to the CredibleMeds^®^ QT-drug lists of the Arizona Center for Education and Research on Therapeutics (AzCERT).^1^ This number has been increasing over the years as new drugs are added to the QT-drug list due to monthly reviews of AzCERT.^2^ QTc-prolongation is used as a surrogate marker for the risk of TdP, a ventricular tachycardia which may ultimately lead to ventricular fibrillation or sudden cardiac death.^3–5^ Although QTc-prolongation is not the perfect marker for arrhythmia risk as many other risk factors play a role in developing TdP, it has become the primary safety parameter among health care professionals, because it is still the most validated marker for the proarrhythmic potency of drugs.^5–7^

Although a QTc-prolonging drug in itself will rarely induce clinically relevant QTc-prolongation (>500 ms), a combination of QTc-prolonging drugs in patients with multiple risk factors can result in QTc-intervals above 500 ms.^8,9^

The Dutch drug database ‘G-standard’, which contains information for clinical decision support, describes the current guidelines for risk management of drug safety alerts. In The Netherlands, clinical decision support (CDS) systems in primary and secondary care generate QT-DDI alerts when two QTc-prolonging drugs with a known risk of TdP are combined. More than 40% of the processed drug prescriptions lead to drug safety alerts.^10^ However, the specificity of the alerts generated by CDS systems is very low, resulting in a low number of interventions. At the moment, there is a complete lack of discrimination when handling these QT-DDIs. Many of the generated QT-DDI alerts do not require an intervention. With the increasing number of QTc-prolonging drugs, alert fatigue could be imposed on physicians. Low specificity alerts contribute to non-compliance with current guidelines.^11–15^ To decrease the alert burden, more advanced clinical rules including clinical parameters such as patient characteristics and laboratory values are used to improve the specificity of the alerts and decrease the alert rate.^16–20^

In the case of QT-DDI alerts, an advanced clinical rule should be able to discriminate low- and high-risk patients for developing QTc-prolongation. QT-DDI alerts are redundant in patients with no other risk factors for QTc-prolongation. In high-risk patients, QTc-prolonging drugs should be either substituted or routine electrocardiogram (ECG) monitoring is required. This clinical rule should be developed irrespective of the QTc-prolonging drugs involved in the QT-DDIs, due to insufficient data on the absolute effect on QTc-prolongation of these drugs. A risk profile of each individual patient will improve accuracy of QT-DDI alerts and support the risk management of QT-DDIs.^8^

**Figure 1.**
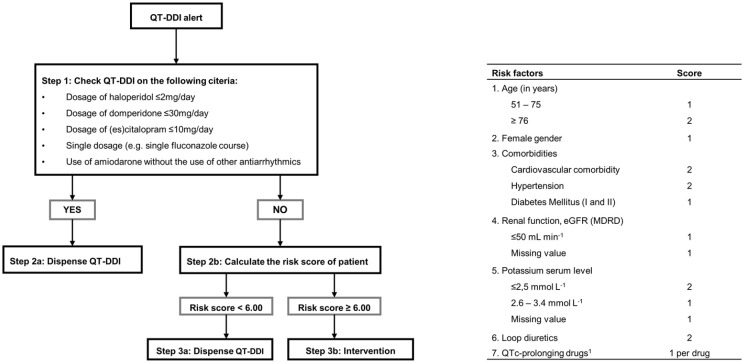
Clinical decision support tool to assess the risk of QT-DDIs. QTc-prolonging drugs with a known risk of TdP^1^ eGFR, estimated glomerular filtration rate; MDRD, Modification of Diet in Renal Disease; QT-DDI, QT drug–drug interaction.

Therefore, a CDS prediction tool was developed to assess the risk of QT-DDIs for developing QTc-prolongation (Figure 1).^21,22^ The main aim of this study was to determine the effect of such a CDS tool on the interventions made by pharmacists in primary care. We also explored the usability of the CDS tool in clinical practice.

## Methods

### Development of a prediction model

This CDS prediction model was developed by performing a prospective observational study in 107 patients using two or more QTc-prolonging drugs with a known risk of TdP^1^ to identify risk factors for QTc-prolongation.^21^ A standard 12-lead resting ECG was recorded at the estimated time of peak concentration (*T*_max_) of the last added drug, or at the longest *T*_max_ in the case of both drugs being started at the same time. Risk factors were identified using logistic regression analyses and risk points were assigned based on the odds ratios. Additional risk factors were incorporated into the model based on a literature review on risk factors for QTc-prolongation. The CDS tool was validated in an external dataset (*n* = 8,453), resulting in an area under the receiver operating characteristic-curve of 0.59 (95% CI 0.54–0.63) when QTc-prolongation was defined as >500 ms due to many false positive results. The selected optimal cut-off value was 6; 26.3% of all patients scored <6 points. A sensitivity of 83.9% and a specificity of 27.5% were accomplished with a cut-off value of 6. The discriminative ability of the tool is not perfect, so optimization of the tool is required.^22^ On the other hand, there is currently a complete lack of discrimination when handling these QT-DDIs. There should be a balance between the number of alerts generated by the CDS systems and its effect on patient care. Although not perfect, this tool is still able to reduce the number of redundant QT-DDI alerts.

### Study design

An intervention study was performed using a pre- and post-design in 20 community pharmacies in The Netherlands. We implemented the use of the CDS tool (consisting a paper-based flowchart) to study the impact on the handling of QT-DDIs.

All QT-DDIs that occurred during a pre- and post-CDS tool period of 3 months were included. The QTc-prolonging drugs involved in the QT-DDIs are listed at the CredibleMeds^®^ QT-drug list with a known risk of TdP (Supplemental material Table S1 online). Only pharmacies using the pharmacy information system Pharmacom^®^ (by TSS Pharma Partners) in the region of Rotterdam (Rijnmond) were included to ensure conformity in data capture and data extraction. The medical ethics review board of the Erasmus University Medical Centre approved the protocol and waived the requirement for obtaining informed consent (MEC-2015-513). The study was conducted according to the principles of the Declaration of Helsinki.

### Study population

All QT-DDIs including QTc-prolonging drugs with a known risk of TdP that occurred in the community pharmacies during a study period of 3 months before and 3 months after implementation of the CDS tool were included. QT-DDIs of patients younger than 18 years old were excluded.

### Outcome measures

The primary outcome measure of this study was the proportion of QT-DDIs in which pharmacists intervened. An intervention was defined as a consultation with prescribers to discuss the clinical relevance of the QT-DDI, and proposal for further actions to be taken (hereafter referred to as intervention). Secondary outcome measures were the types of interventions made by pharmacists. The interventions for the QT-DDIs were subsequently categorized in (I) dispensing both drugs on account of the prescriber, (II) ECG monitoring, (III) substitution of one of the interacting agents, (IV) dose adjustments or (V) (temporarily) stopping one of the interacting agents. We have also studied the difference between first-time prescriptions and repeat-prescriptions on the handling and types of interventions. Another secondary outcome measure was the time spent on handling QT-DDIs. Finally, the CDS tool was evaluated by pharmacists on usability in clinical practice.

### Data collection

For all QT-DDIs, the following variables were collected: the management of the QT-DDI including interventions, the interacting drugs and the dosages of interacting drugs.

For all patients: age, gender, comorbidities (registered as drug–disease interactions) were collected. Concomitant drug use was retrieved from the medication history up to 1 year prior to the QT-DDI alert. The following laboratory values were collected, if registered in Pharmacom^®^: renal function, liver function parameters and electrolyte serum levels. Patient data were handled confidentially and were extracted anonymously according to the Dutch Personal Data Protection Act (Wbp). All patients with QT-DDI alerts were captured in an electronic clinical data management system (OpenClinica, LLC, Waltham, United States).

### CDS tool

The CDS tool was implemented in the participating community pharmacies after a 3 month baseline analysis as a tool to support the electronic handling of QT-DDI alerts. The CDS tool consisted of a paper-based flowchart identifying patients that were at increased risk for developing QTc-prolongation as is shown in Figure 1. The criteria of step 1 of the flowchart were based on a literature review and an expert panel with two cardiologists with expertise in electrophysiology and publications in the field.^23–30^ Before implementation, the pharmacists were trained in using the CDS tool. The CDS tool was expected to be used for all QT-DDI alerts during the post-implementation period. When patients scored ⩾6 using the tool, an intervention by pharmacists was recommended. The risk scores and types of interventions were documented using a paper form.

### Usability clinical decision support tool

After a period of 3 months, the tool was evaluated by the pharmacists on usability in clinical practice using the System Usability Scale (SUS) of Brooke.^28,29^ The SUS is based on 3 usability measures suggested by the International Organization for Standardization (ISO)-9241-11: effectiveness, efficiency and satisfaction. The SUS consists of a 10-item questionnaire covering subjective items of usability using a five-point Likert scale with a degree from total disagreement (1) to total agreement (5). For evaluation of the CDS tool, 10 items were formulated so that they were compatible for the CDS tool (Supplemental Table S2). Items 1, 3, 6, 7 and 9 were positively formulated and items 2, 4, 5, 8 and 10 were negatively formulated. The SUS score was calculated, first by recalculating the score of each item (1–5) to a range from 0–4 using the following formula; for the positively formulated items: scale position minus 1, and for the negatively formulated items: 5 minus scale position. Second, the sum of these scores was multiplied by 2.5 to obtain the overall SUS score.^31^ Total SUS scores range from 0 to 100; if SUS scores are <60 the system is considered to be unacceptable, 60–70 is acceptable, 70–80 is good, 80–90 is very good and >90 is excellent.^32,33^

### Statistical analysis

Based on information provided by the Stevens Institute for Research (SIR) in Leiden, The Netherlands, Dutch community pharmacies on average dispense QTc-prolonging drugs 140 times a year with a QT-DDI (interaction code 6297). That results in 0.5 QT-DDIs per day per pharmacy in which the DDI-causing drug is dispensed. A QT-DDI does not always result in dispensing, so we estimate that one QT-DDI per day per pharmacy will be handled. So, in 3 months (pre and post measurement) approximately 45 QT-DDIs per pharmacy were expected to be monitored, which would result in a total number of 900 QT-DDIs in 20 community pharmacies. With an alpha of 0.05 and a power of 80%, a difference in proportion of QT-DDIs with intervention of 60% (pre) *versus* 66% (post) can be established using logistic regression, estimating that in 60% of the QT-DDIs an intervention is carried out.

The primary outcome was determined by dividing the number of QT-DDIs with an intervention by the total number of QT-DDIs. Univariate logistic regression analysis was used to analyse the primary outcome between the measurements before and after implementation of the CDS tool. If a variation in interaction characteristics occurred between the before- and after-period, the primary outcome was adjusted using multivariate logistic regression analysis. Odds ratios (ORs) and their 95% confidence intervals (CIs) were reported. Secondary outcome measures were analysed using descriptive statistics.

## Results

### Study population

**Figure 2.**
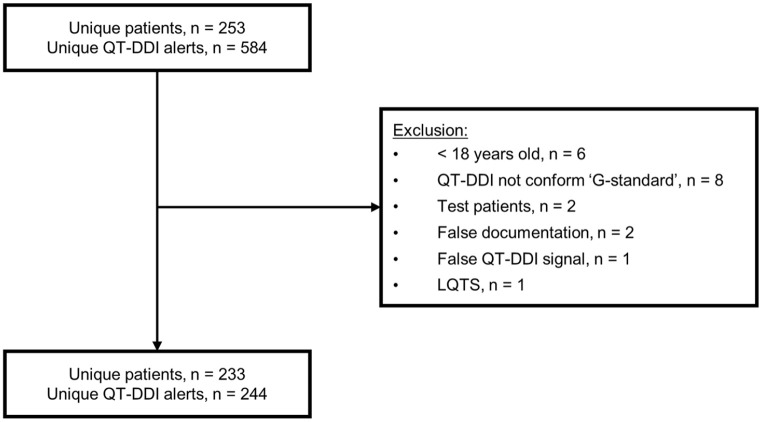
Flowchart of QT-DDI inclusions in before-period. LQTS, long QT syndrome; QT-DDI, QT drug–drug interaction.

**Figure 3.**
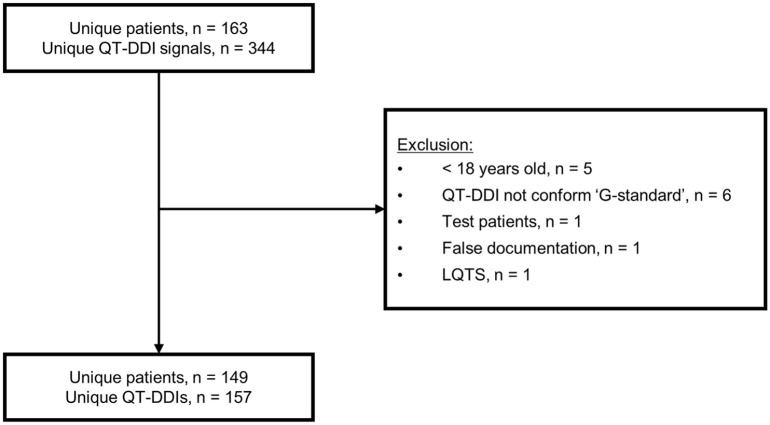
Flowchart of QT-DDI inclusions in after-period. LQTS, long QT syndrome; QT-DDI, QT drug–drug interaction.

The baseline characteristics of the participating community pharmacies are presented in Table 1. The after-period included 16 community pharmacies because four pharmacists, and therefore four pharmacies, discontinued their participation because of construction work of the pharmacy and shortages in personnel. A total of 928 QT-DDI alerts were generated during the pre- and post-CDS tool phases, of which 401 QT-DDIs were included for analysis. In the before-period, 244 QT-DDIs belonging to 233 patients were included for analysis. Six QT-DDIs were excluded because they occurred in patients <18 years old (Figure 2). In the after-period, a total of 157 QT-DDIs of 149 patients were included, as shown in Figure 3. Only 23 patients were included in both the before- and after-periods; in 17 patients the QT-DDIs were identical.

**Table 1.** Baseline characteristics of the participating community pharmacies.

Pharmacy characteristics	Cohort *N* = 20
FTE pharmacists, mean ± SD	1.7 ± 0.9
FTE pharmacy assistants, mean ± SD	7.0 ± 3.3
HKZ certificates, *n* (%)	
None	−
Chain certificate	7 (35)
Own certificate	13 (65)
Collaboration with other community pharmacies, *n* (%)	
None	8 (40)
<5	4 (20)
5–25	3 (15)
>25	5 (25)
GPs responsible for >80% of prescriptions, *n* (%)	
1–3	5 (25)
4–6	10 (50)
7–9	3 (15)
⩾10	2 (10)
Shared patient records with GP, *n* (%)	14 (70)
Community Health Centre, *n* (%)	12 (60)
Use of EPR in Pharmacom^®^, *n* (%)	18 (90)
% of renal function parameters available, mean ± SD	71.7 ± 20.9
% of potassium serum levels available, mean ± SD	58.1 ± 31.9
% of shared contra-indications with GP, mean ± SD	77.2 ± 19.7

EPR, electronic patient record; FTE, fulltime-equivalent; GP, general practitioner; HKZ, Harmonization quality assessment in Health Sector; SD, standard deviation.

The baseline patient characteristics of patients in the before- and after-periods are shown in Table 2. The two groups did not significantly differ in patient characteristics. The median (interquartile range) age of the total cohort was 65 (28) years and most QT-DDI alerts belonged to female patients (64.1%). From only a limited numbers of patients the renal function parameters (10.2%) and potassium levels (0.8%) could be extracted from Pharmacom^®^. In clinical practice, pharmacy information systems are frequently linked to the information system of the general practitioners (GPs). Unfortunately, it was not possible to retrieve these variables from the GP information system due to privacy legislation.

**Table 2.** Baseline characteristics of study population.

Patient characteristics	Before-period	After-period	*p*-value
	*n* = 233	*n* = 149	
Age, years, median; IQR	66.0; 26.0	63.0; 31.0	0.86^a^
⩽50, *n* (%)	60 (25.8)	41 (27.5)	0.72^b^
51–75, *n* (%)	113 (48.5)	66 (44.3)	
⩾76, *n* (%)	60 (25.8)	42 (28.2)	
Female gender, *n* (%)	154 (66.1)	91 (61.1)	0.32^b^
BMI, median; IQR	31.9 (*n* = 1)	24.5 (*n* = 1)	−
Comorbidities, *n* (%)			
Myocardial infarction	25 (10.7)	17 (11.4)	0.84^b^
Heart failure	13 (5.6)	2 (1.3)	0.06^c^
Arrhythmia	14 (6.0)	3 (2.0)	0.08^c^
Hypertension	68 (29.2)	43 (28.9)	0.95^b^
Diabetes mellitus	39 (16.7)	29 (19.5)	0.50^b^
COPD/asthma	42 (18.0)	23 (15.4)	0.51^b^
CVA/TIA	5 (2.1)	8 (5.4)	0.09^b^
Renal dysfunction	16 (6.9)	12 (8.1)	0.66^b^
Liver dysfunction	9 (3.9)	4 (2.7)	0.77^c^
Others	111 (47.6)	74 (49.7)	0.70^b^
Renal dysfunction with renal function, *n* (%)			
eGFR, MDRD ⩽50 ml/min	11 (4.7) (*n* = 20)	10 (6.7) (*n* = 19)	0.97^b^
Electrolyte disturbances, *n* (%)			
Hyponatraemia (Na^+^ <136 mmol/l)	− (*n* = 1)	− (*n* = 2)	−
Hypokalaemia (K^+^ <3.5 mmol/l)	− (*n* = 1)	− (*n* = 2)	−

a Independent *t* test.

b Chi-square test.

c Fisher’s exact test.

BMI, body mass index; COPD, chronic obstructive pulmonary disease; CVA, cerebrovascular accident; eGFR, estimated glomerular filtration rate; IQR, interquartile range; MDRD, Modification of Diet in Renal Disease; TIA, transient ischemic attack.

There was no significant difference in the proportion of first-time prescriptions in the QT-DDIs in the before-period (62.5%) compared with the after-period (73.9%, *p* = 0.08). In both periods, the QT-DDI that occurred most frequently was (es)citalopram – haloperidol (10.8% and 10.7%, respectively). The top-10 QT-DDIs and QTc-prolonging drugs are presented in Table 3. When drug groups were classified, the most common QT-DDI was a QTc-prolonging drug combined with an antiarrhythmic agent class III (34.0%) in the before-period and a QTc-prolonging drug combined with a QTc-prolonging antibiotic (32.5%) in the after-period. The mean ± SD number of QT-DDIs per pharmacy was 12.2 ± 7.6 in the before-period and 9.8 ± 5.1 in the after-period.

**Table 3.** Top-10 QT-DDIs.

QT-DDIs	Before-period	After-period
	*n* = 244 (%)	*n* = 157 (%)
1. Haloperidol – (es)citalopram	26 (10.7)	17 (10.8)
2. Amiodarone – ciprofloxacin	13 (5.3)	7 (4.5)
3. Azithromycin – (es)citalopram	12 (4.9)	13 (8.3)
4. (Es)citalopram – fluconazole	11 (4.5)	5 (3.2)
5. Azithromycin – domperidone	10 (4.1)	2 (1.3)
6. Sotalol – ciprofloxacin	10 (4.1)	5 (3.2)
7. Haloperidol – ciprofloxacin	9 (3.7)	7 (4.5)
8. Sotalol – flecainide	8 (3.3)	6 (3.8)
9. Sotalol – (es)citalopram	7 (2.9)	−
10. Azithromycin – fluconazole	6 (2.4)	10 (6.4)

Missing values 1 and 2.

QT-DDI, QT drug–drug interaction.

### Outcome measures

There was no significant difference in the proportion of QT-DDIs for which an intervention was made after implementing the CDS tool; 43.0% before and 35.7% after implementation (OR 0.74, 95% CI 0.49–1.11; *p* = 0.14). A sensitivity analysis, performed by excluding 23 patients who were included in both the pre- and the post-period, had no effect on the significance of the primary outcome (OR 0.78, 95% CI 0.50–1.21; *p* = 0.27).

An interacting agent was substituted in 41.0% of the interventions in the before-period and in 46.4% in the after-period. In 37.7% of the QT-DDIs with intervention, the QTc-prolonging drugs were dispensed in the before-period, and in 26.8% of the QT-DDIs in the after-period. Almost 12% of all alerts were incorrect QT-alerts because one of the interacting agents was already stopped or the combination was not given simultaneously. In 5.2% of the QT-DDIs, pharmacists did not properly document their actions. The variety of interventions performed by pharmacists are presented in Table 4.

**Table 4.** Type of intervention.

Interventions	Before-period	After-period	*p*-value
	*n* = 244 (%)	*n* = 157 (%)	
Dispensed on account of prescriber	30 (12.3)	12 (7.6)	
Substitution of one of the interacting agents	43 (17.6)	26 (16.6)	
(Temporarily) stopping one of the interacting agents	23 (9.4)	15 (9.6)	
Dose adjustments	4 (1.6)	3 (1.9)	
ECG monitoring	5 (2.0)	-	
**Total**	**105 (43.0)**	**56 (35.7)**	**0.14**

ECG, electrocardiogram.

After implementation, 157 QT-DDI alerts were handled electronically. However, pharmacists completed the paper forms in only 30.6% of the QT-DDI alerts. Of these, 30 QT-DDIs (63.5%) could be dispensed without intervention according to the flowchart of the CDS tool. Pharmacists dispensed the QT-DDI with no intervention, and therefore adhered to the flowchart in 19 cases (63.3%). In the remaining cases (*n* = 11), prescribers were consulted. Note that six (54.5%) of these cases included amiodarone, of which five (83.3%) had a risk score ⩾6; in these cases, an intervention was performed. Of the 18 QT-DDIs where the flowchart advised to score patients using the risk model, four patients scored <6; these QT-DDIs were dispensed without intervention. In all patients with a risk score ⩾6, prescribers were consulted and in 92.8% an intervention was performed. Taking all QT-DDIs into account, the overall compliance with the CDS tool by pharmacists was 75%.

When we focus on the criteria of step 1 of the flowchart, the proportion of interventions when QT-DDIs included amiodarone did not differ between the two periods (48.1% *versus* 47.6%; *p* = 0.97). The proportion of interventions decreased in QT-DDIs including (es)citalopram ⩽10 mg (42.3% *versus* 17.3%; *p* = 0.07), domperidone ⩽30 mg (45.9% *versus* 9.1%; *p* = 0.03) and haloperidol ⩽2 mg (35.7% *versus* 20.6%; *p* = 0.15). Only in the case of domperidone, the proportion of interventions decreased significantly between the before- and the after-period. The difference in types of interventions for QT-DDIs including first-time prescriptions or repeat-prescriptions is shown in Table 5.

**Table 5.** Intervention rate of alerts divided into first-time (FP) and repeat (RP) prescriptions.

	Before-period		After-period	
	Intervention (%)	No intervention (%)	Intervention (%)	No intervention (%)
FP	87 (57.2)	65 (42.8)	47 (41.2)	67 (58.8)
RP	18 (19.6)	74 (80.4)	9 (20.9)	34 (79.1)
*p*-value^a^	<0.001		0.018	

a Chi square test.

#### Time spent by pharmacists

In the before-period, nine pharmacists documented the time spent on the management of QT-DDIs in 56 QT-DDIs. On average, pharmacists spent 21 min on the management of QT-DDIs, as shown in Table 6. Consultation with the prescriber was the most time-consuming and took 10 min on average. In five cases the handling of QT-DDIs encompassed more than 1 day, because the pharmacists could not reach the prescriber. Nevertheless, these days were not counted in the overall time as this does not represent the time pharmacists actually spent on QT-DDIs.

**Table 6.** Time for handling QT-DDIs in community pharmacies before implementation of the clinical decision support tool.

Time-management QT-DDIs	FP, *n* = 38		RP, *n* = 12		Discharge, *n* = 5	
	*n* (%)	Minutes Mean ± SD	*n* (%)	Minutes Mean ± SD	*n* (%)	Minutes Mean ± SD
Literature	19 (50.0)	2.6 ± 4.4	3 (25.0)	0.9 ± 1.7	4 (80.0)	10.4 ± 11.9
Consult prescriber	26 (68.4)	12.3 ± 26.8	2 (16.7)	1.0 ± 2.4	3 (60.0)	11.0 ± 16.7
Consult PA	18 (47.4)	1.8 ± 2.5	6 (50.0)	2.8 ± 3.5	3 (60.0)	2.6 ± 4.2
Documentation in PIS	32 (84.2)	2.6 ± 3.7	7 (58.3)	2.8 ± 3.8	5 (100.0)	5.8 ± 4,.1
Consult patient	23 (60.5)	2.5 ± 3.2	3 (25.0)	2.8 ± 8.6	2 (40.0)	3.2 ± 6.6
Other	6 (15.8)	0.8 ± 2.1	2 (16.7)	0.2 ± 0.4	−	−
**Total, mean ± SD**		**22.6 ± 28.8**		**10.8 ± 11.1**		**33.0 ± 34.2**

FP, first-time prescription; PA, pharmacy assistant; PIS, Pharmacy Information System; QT-DDI, QT drug–drug interaction; RP, repeat prescription.

After implementation, time management was documented in 48 QT-DDIs (Table 7). Pharmacists selected a variety of QT-DDIs. Based on these QT-DDI, pharmacists spent on average 15 min on the management of QT-DDIs, approximately 6 min less than in the before-period. Time spent on consultation with the prescriber was reduced to 4 min. Of these 15 min, nearly 5 min were spent on completing the CDS flowchart.

**Table 7.** Time for handling QT-DDIs in community pharmacies after implementation of the clinical decision support tool.

Time-management QT-DDIs	FP, *n* = 40		RP, *n* = 4		Discharge, *n* = 1	
	*n* (%)	Minutes Mean ± SD	*n* (%)	Minutes Mean ± SD	*n* (%)	Minutes Mean ± SD
Literature	−	−	−	−	−	−
Consult prescriber	23 (57.5)	4.5 ± 6.1	−	−	−	−
Consult PA	17 (42.5)	1.3 ± 2.3	2 (50.0)	0.7 ± 0.6	1	4.0
Documentation in PIS	36 (90.0)	2.0 ± 2.0	3 (75.0)	3.0 ± 2.0	1	2.0
Consult patient	17 (42.5)	1.8 ± 3.4	1 (25.0)	0.3 ± 0.6	1	10.0
Other	8 (20.0)	1.7 ± 4.1	1 (25.0)	0.7 ± 1.2	1	3.0
Clinical rule	37 (92.5)	4.4 ± 4.5	3 (25.0)	7.0 ± 6.9	1	1.0
**Total, mean ± SD**		**15.0 ± 16.3**		**11.7 ± 7.6**		**20.0**

FP, first-time prescription; PA, pharmacy assistant; PIS, Pharmacy Information System; QT-DDI, QT drug–drug interaction; RP, repeat prescription.

#### Usability CDS prediction tool

The mean ± SD SUS score of the CDS tool was 74.1 ± 19.1 (14 pharmacists). The highest (maximum of 4) mean score per question was 3.5 and was scored on question 1 regarding satisfaction; pharmacists would like to use the CDS tool in clinical practice. The lowest (minimum of 0) mean score per question was scored on question 4 regarding reliability; pharmacists expected to need the support of different literature sources, besides the tool, to safely manage QT-DDIs. Three pharmacists ranked the CDS tool <60 and considered the tool as ‘unacceptable’, mainly because the tool was time-consuming in their opinion. The overall suggestion of most pharmacists was to eventually integrate the clinical decision support tool into the pharmacy information system.

## Discussion

To our knowledge, this was the first study on a CDS tool to support the handling of QT-DDIs in community pharmacies. Our study has shown that the implementation of an advanced CDS tool did, not significantly, reduce the proportion of QT-DDIs for which an intervention was made (43.0–35.7%, *p* = 0.14). However, pharmacists seemed to spend less time on the management of QT-DDIs when the CDS tool was used (6 min per QT-DDI). Overall, the pharmacists were satisfied using the tool to support the management of QT-DDIs in clinical practice.

At first, we hypothesized that an advanced CDS tool would result in more interventions, but more than 70% of the QT-DDI alerts did not require an intervention according to the CDS tool and could be considered as irrelevant. Several other studies show that specification of alert triggers by advanced clinical decision rules could decrease the alert rate up to 90%.^34–37^ In total, 75% of QT-DDIs were handled with the CDS tool. Pharmacists did not comply with the flowchart of the CDS tool when the QT-DDIs included amiodarone, because they did not feel comfortable dispensing QT-DDIs with amiodarone. Therefore, if the tool is implemented in clinical practice, education on the risks of QTc-prolongation and TdP can be useful to achieve more compliance by pharmacists.

Repeat prescriptions for chronic medication are common in primary care. However, many QT-DDIs are only relevant at the start of therapy, and are, therefore, more likely to be followed by an intervention.^11^ In our study, 50% of the first-time prescription QT-DDIs were followed by an intervention and 21% of the repeat-prescription QT-DDIs were followed by an intervention, as is shown in Table 7. The CDS tool enables reassessment of repeat-prescription QT-DDIs when the condition of a patient might change during chronic treatment where a patient might become a high-risk patient.

Van der Sijs *et al.* showed that QT-DDI overriding rarely (33%) results in ECG recording in a hospital setting.^38^ Expectedly, our study showed that in primary care this percentage is even lower (1.7%), as ECG recording is not feasible in community pharmacies. The CDS tool does not specify management recommendations when an intervention is required, as an individualized risk assessment depending on the patient’s situation by a health care professional is still important to determine the type of intervention. The combination of QTc-prolonging drugs may result in potential fatal TdP, which rarely occurs. Consensus exists that measuring the QTc-interval on the ECG is the best option to predict which patients are at risk, and the QTc is a proxy for the patient outcome. However, making ECGs for all patients filling their prescriptions in community pharmacies is not feasible and not necessary, because the QTc-prolongation risk may vary considerably among patients and QT-DDIs do not always require intervention.

A strength of this study was the pre- and post-design of the study which enables us to study the current handling of QT-DDIs. Also, the 20 community pharmacies included in this study represent the general Dutch community pharmacies according to the Dutch Foundation for Pharmaceutical Statistics (SFK) Facts and Figures of 2015.

This study also has some potential limitations. First, the tool was paper-based and was not integrated into the electronic CDS system of the community pharmacies. Consequently, the documentation of the QT-DDIs was limited because work processes in community pharmacies are fully digitalized. Only 48 forms were completed *versus* the 157 unique QT-DDIs generated by the CDS system in the after-period. We do realize that due to these limited completed forms our study was underpowered to make definitive conclusions regarding the tool. We hypothesize that the non-significant decrease in interventions in this study might turn into a significant decrease in interventions when performed in a larger dataset. Thereby, it should be noticed that the discriminative ability of the tool was poor, and missed errors may have occurred.

Second, apart from the criteria incorporated in step 1 of the flowchart, the tool does not stratify the various QT-DDIs. Although the QTc-prolonging drugs have different pharmacological pathways for inducing QTc-prolongation, it is relatively unknown whether combining QTc-prolonging drugs with different pharmacological pathways has an additive or synergistic effect on the extent of QTc-prolongation.^39,40^ Therefore, in this study we assumed the synergistic effect of the QTc-prolonging drugs to be similar. Also, the number of laboratory values retrieved from the pharmacy information systems was low. In reality, this number of available laboratory values is higher because pharmacy information systems are frequently linked to information systems of GPs. In 58.1% of the included pharmacies, the pharmacy information system was linked to the GP information system, as shown in Table 1. Additionally, a potassium level of <2.5 mmol L^−1^ scores two points according to the risk model; however, it is unlikely that an outpatient would have a value this low and any measurement this low would likely have been managed in an inpatient setting. Although the aim of the present study was to evaluate how such a tool would perform in a primary care setting, the tool was also developed for use in hospitals, and in such a setting a low potassium level may occur.

In total, four pharmacies dropped out of the study due to construction work of the pharmacy and shortages of personnel. These could potentially lead to biased results, but as these pharmacists dropped out before the implementation of the tool, this is probably not the case.

For future perspectives, this clinical decision support tool deserves further investigation to assess its effect when it is integrated in the pharmacy information system. Such a study should be performed in large patient groups with clinically relevant endpoints such as QTc-prolongation before implementation in clinical practice can be recommended. Ideally, the system will then automatically calculate a risk score for the individual patient and only generate alerts if the risk score is >6, resulting in more specific alerts.

In conclusion, these results suggest that the clinical decision support tool might be an effective tool to manage QT-DDIs *via* a structured approach, through which a more specific advice can be given to prescribers. Also, if the condition of patients were to change during chronic treatment, the CDS tool can easily identify these potential harmful changes. Pharmacists are satisfied to use the tool and it has proven to be feasible in clinical practice. However, optimization of the tool is required before implementation in clinical practice.

## Supplemental Material

sj-pdf-1-taw-10.1177_2042098621996098 – Supplemental material for The use of a clinical decision support tool to assess the risk of QT drug–drug interactions in community pharmacies Click here for additional data file. Supplemental material, sj-pdf-1-taw-10.1177_2042098621996098 for The use of a clinical decision support tool to assess the risk of QT drug–drug interactions in community pharmacies by Florine A. Berger, Heleen van der Sijs, Teun van Gelder and Patricia M. L. A. van den Bemt in Therapeutic Advances in Drug Safety
