# Supplementary material for: The use of a clinical decision support tool to assess the risk of QT drug–drug interactions in community pharmacies
Source: Ther Adv Drug Saf. 2021 Feb 24;12:2042098621996098. doi: 10.1177/2042098621996098 (PMC7907715; doi:10.1177/2042098621996098)
Supplement: sj-pdf-1-taw-10.1177_2042098621996098 – Supplemental material for The use of a clinical decision support tool to assess the risk of QT drug–drug interactions in community pharmacies [file sj-pdf-1-taw-10.1177_2042098621996098.pdf]

Supplementary material

Table S1 – QTc-prolonging drugs with a known risk of TdP

ATC-code	Drugs
C01BD01	Amiodarone
A02BD04	amoxicillin/clarithromycin/pantoprazole
L01XX35	Anagrelide
L01XX27	arsenic trioxide
J01FA10	Azithromycin
N05AA01	Chlorpromazine
P01BA01	Chloroquine
J01MA02	Ciprofloxacin
N06AB04	Citalopram
J01FA09	Clarithromycin
C01BA03	Disopyramide
A03FA03	Domperidone
N06DA02	Donepezil
N05AD08	Droperidol
J01FA01	Erythromycin
N06AB10	Escitalopram
C01BC04	Flecainide
J02AC01	Fluconazole
N05AD01	Haloperidol
C01BD05	Ibutilide
C02KD01	Ketanserin
C01BA01	Quinidine
J01MA12	Levofloxacin
N05AA02	Levomepromazine
N07BC02	Methadone
J01MA14	Moxifloxacin
A04AA01	Ondansetron
L01XA03	Oxaliplatin
A03AD01	Papaverine
G04BE30	papaverine/phentolamine
P01CX01	Pentamidine
N05AG02	Pimozide
C01BA02	Procainamide
N01AX10	Propofol
J01FA06	Roxithromycin
N01AB08	Sevoflurane
C07AA07	Sotalol
N05AL01	Sulpiride
H01BA04	Terlipressin
L01XE12	Vandetanib

Table S2 – Questionnaire evaluation clinical rule

System Usability Scale (SUS)*						
		Totally disagree				Totally agree
1	I think that I would like to use this clinical rule frequently.	○ 1	○ 2	○ 3	○ 4	○ 5
2	I found the clinical rule unnecessarily complex.	○ 1	○ 2	○ 3	○ 4	○ 5
3	I thought the clinical rule was easy to use.	○ 1	○ 2	○ 3	○ 4	○ 5
4	I think that I would need the support of different literature sources, besides the clinical rule, to handle QT-interactions.	○ 1	○ 2	○ 3	○ 4	○ 5
5	I quickly make mistakes with the calculation and method of the clinical rule.	○ 1	○ 2	○ 3	○ 4	○ 5
6	I found the consecutive steps of the clinical rule logical.	○ 1	○ 2	○ 3	○ 4	○ 5
7	I would imagine that most people would learn to use this clinical rule very quickly.	○ 1	○ 2	○ 3	○ 4	○ 5
8	I found the clinical rule difficult to use.	○ 1	○ 2	○ 3	○ 4	○ 5
9	I felt very confident using the clinical rule.	○ 1	○ 2	○ 3	○ 4	○ 5
10	I needed to learn a lot of things before I could get going with the clinical rule.	○ 1	○ 2	○ 3	○ 4	○ 5
11	What would you suggest to optimize the usability of this clinical rule?					

*System Usability Scale (SUS), Brooke JS, 1996
